# Mixed Intervillositis in SARS-CoV-2 Infection Associated with Fetal Death: A Case Report

**DOI:** 10.1155/2022/5404952

**Published:** 2022-12-02

**Authors:** Boban Gjakov, Darja Kopač, Mateja Vukmanič Pohar, Miha Lučovnik

**Affiliations:** ^1^Department of Gynecology and Obstetrics, Brežice General Hospital, Černelčeva cesta 15, 8250, Brežice, Slovenia; ^2^Division of Obstetrics and Gynecology, University Medical Center Ljubljana, Šlajmerjeva 3, 1000 Ljubljana, Slovenia; ^3^Medical Faculty, University of Ljubljana, Vrazov trg 2, 1000 Ljubljana, Slovenia

## Abstract

Maternal severe acute respiratory syndrome coronavirus 2 (SARS-CoV-2) infection has been shown in epidemiological studies to be associated with an increased risk of stillbirth. Several histopathologic features of placental SARS-CoV-2 infection have been proposed as potential causes of fetal death. We present a case of an otherwise healthy G3P1 women with mild symptoms of SARS-CoV-2 infection at 23 6/7 weeks of gestation. At 25 2/7 weeks, she presented with signs and symptoms of preterm labor and decreased fetal movements. Fetal death was diagnosed at admission. Placental pathology showed pronounced placental mixed intervillositis. Inflammatory infiltrate caused extreme narrowing of intervillous space leading to placental malfunction and fetal death. Placental tissue SARS-CoV-2 infection was confirmed by positive immunohistochemistry staining of syncytiotrophoblasts with spike protein antibody. The case presented suggests that SARS-CoV-2 associated inflammatory placental changes pose an elevated risk for the fetus.

## 1. Introduction

Pregnant women are at increased risk of severe and critical coronavirus disease 2019 (COVID-19) due to immunological and cardiopulmonary changes related to pregnancy [1, 2]. They are more likely to require intensive care unit admission, invasive mechanical ventilation, and extracorporeal membrane oxygenation than nonpregnant severe acute respiratory syndrome coronavirus 2 (SARS-CoV-2) positive women of reproductive age [1–5]. COVID-19 has also been associated with increased risk of maternal mortality [1, 6].

In addition to the impact of pregnancy on COVID-19 severity, there are also concerns of adverse effects of SARS-CoV-2 infection during pregnancy on the fetus. Maternal COVID-19 has been shown to be associated with an increased incidence of small-for-gestational age neonatal birth weight, preterm birth, and approximately doubled risk of stillbirth [1, 7–10]. Neonatal infections due to vertical SARS-CoV-2 transmission have been infrequently reported, but they can occur under certain biologic conditions [11–13]. Several potential mechanisms that could cause impairment of placental function from COVID-19 infection have been proposed so far. Placental pathology studies have reported increased rates of features associated with maternal and fetal vascular malperfusion in COVID-19 [14–17]. However, most of these were not significantly associated with severity of maternal illness nor with fetal or neonatal outcomes [14–17]. More recently, placental inflammatory infiltrates have been proposed as a feature of acute placental SARS-CoV-2 infection, which could lead to adverse perinatal outcomes [17, 18]. We present a case of fetal death following SARS-CoV-2 infection in the second trimester of pregnancy with pronounced mixed intervillositis seen on placental histopathologic examination. Consent to publish this case report was obtained from the patient.

## 2. Case Presentation

25-year-old G3P1 was diagnosed with SARS-CoV-2 infection (by reverse-transcription polymerase chain reaction test) at 23 6/7 weeks of gestation. She had mild COVID-19 symptoms, i.e., cough, sore throat, and fever (up to 38.5°C) with no dyspnea. She had not been vaccinated against COVID-19 and was otherwise healthy, not taking any medications, with a body-mass-index of 23 kg/m2. She attended regular prenatal visits, had normal first trimester genetic screening, normal second trimester anatomy scan, no signs of gestational diabetes mellitus or hypertensive disorders in pregnancy, and negative toxoplasmosis, hepatitis B, and syphilis screening. Her first trimester indirect Coombs' test was also negative. In her previous pregnancy in 2020, she delivered a 2480 g neonate at 35 weeks of gestation. She had a medical termination of pregnancy at 8 weeks in her second pregnancy.

Ten days after confirmed SARS-CoV-2 infection (at 25 2/7 weeks), she presented at obstetrical triage because she could not feel fetal movements for the last 12 hours. She also reported uterine contractions and vaginal bleeding at that time. Intrauterine fetal death was confirmed by ultrasound. The cervix was 5 cm dilated, and she was admitted to labor and delivery unit, where she delivered a 680 g stillborn neonate. The fetus, umbilical cord, and placenta were send for histopathologic examination.

**Figure 1:**
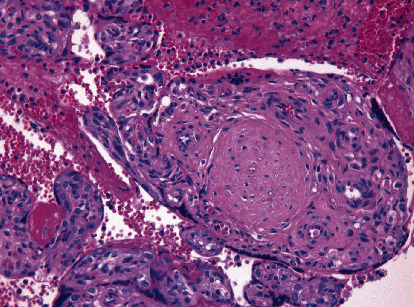
Placental pathology in SARS-CoV-2 infection; haematoxylin and eosin staining. Heavy intervillous acute and chronic inflammatory infiltrates narrow a collapsing intervillous space.

**Figure 2:**
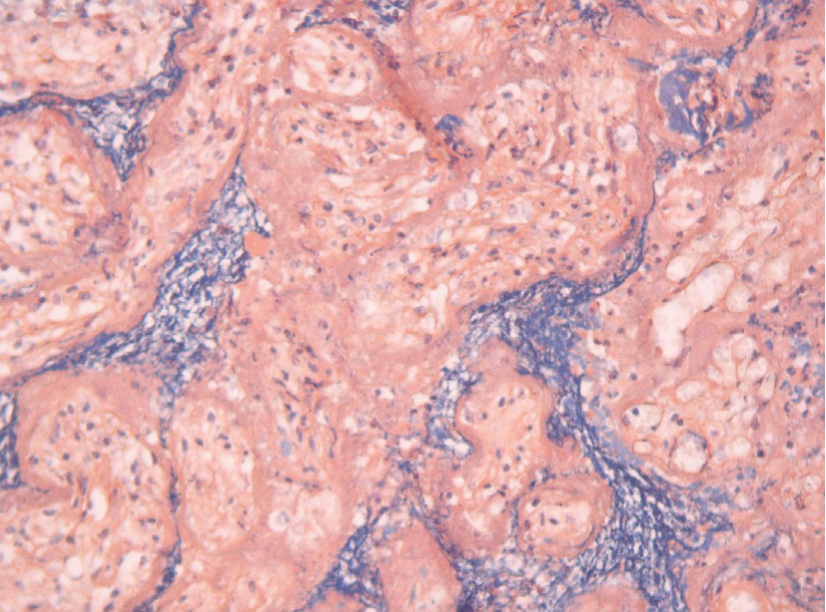
Increased perivillous fibrinoid deposition, phosphotungstic acid haematoxylin (PTAH) stain.

Fetal autopsy found no congenital malformations and no signs of fetal viral infection. There were also no macroscopic signs of acute inflammation in the umbilical cord, which had two arteries and one vein. Placenta weighted 270 g (75 centile for gestational age). On histologic examination, diffuse villous agglutination could be seen accompanied by massive trophoblast necrosis (highlighted by homogenized, hyperchromatic and dusky nuclei that were pycnotic and karyorrhectic at late stages with an often persistent cytoplasmatic clearing). Heavy intervillous acute and chronic inflammatory infiltrates could be observed (Figure 1), admixed with variable amounts of increased perivillous fibrinoid deposition, highlighted by special phosphotungstic acid haematoxylin (PTAH) stain (Figure 2). Extreme narrowing of already collapsing intervillous space by inflammatory infiltrates was seen (Figure 1).

**Figure 3:**
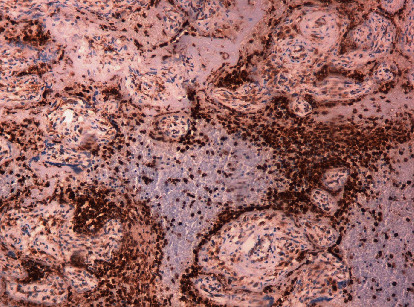
Inflammatory intervillous infiltrate; CD68 positive hystiocytes.

**Figure 4:**
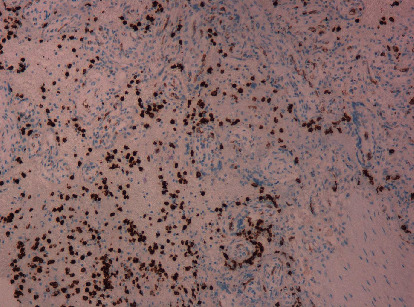
Inflammatory intervillous infiltrate; CD15 positive neutrophils.

The inflammatory infiltrate was composed predominantly of hystiocytes highlighted by immunohistochemistry with strongly positive CD 68 (Figure 3) and neutrophils and positively stained with CD 15 (Figure 4).

**Figure 5:**
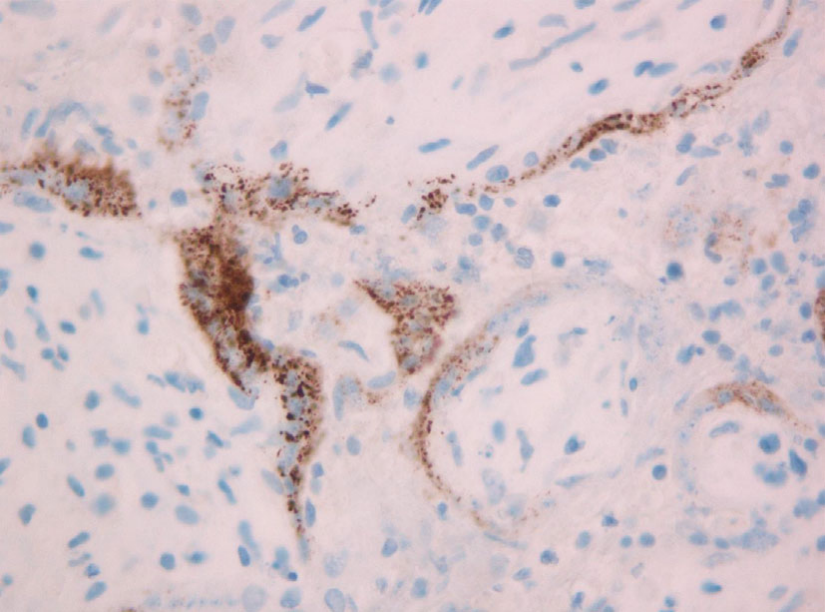
Positive immunohistochemistry staining of syncytiotrophoblasts with spike protein antibody.

Positive immunohistochemistry staining of syncytiotrophoblasts with spike protein antibody showed a granular binding pattern in syncytiotrophoblasts (Figure 5). Villous stroma remained unaffected with a well-preserved capillary network and sporadic local placed nodular intravillous fibrinoid.

## 3. Discussion

We present a case of an intrauterine fetal death at 25 weeks of pregnancy complicated by SARS-CoV-2 infection associated with prominent mixed intervillositis. Reporting placental pathology findings in pregnancies with adverse fetal/neonatal outcomes after maternal COVID-19 is essential to elucidate potential mechanisms that could explain increased rates of perinatal complications reported with this disease, such as fetal growth restriction and stillbirth [1, 7–10].

Placental tissue has already been described to be the target of several viral infections, such as Cytomegalovirus, Herpes simplex virus, Rubella, and more recently Zika, leading to a wide range of placental histopathologic changes [19, 20]. COVID-19 has also been associated with a variegated spectrum of placental pathology. Most early studies reported features of maternal and fetal malperfusion that could result from placental SARS-CoV-2 infection [14–17]. Such features included increase in intervillous fibrin and decidual vasculopathy, thrombosis of fetal microvasculature, delayed villous maturation, and chorangiosis [17]. However, more recent studies did not confirm a significant causal correlation between SARS-CoV-2 infection in pregnancy and an increased incidence of histologic features of malperfusion [21–24]. Moreover, most studies did not explore associations between placental pathology findings and COVID-19 severity, time from infection to delivery, and pregnancy outcomes.

Inflammatory response, such as villitis and intervillositis, was observed much less frequently in placentas following SARS-CoV-2 infection [14–16]. It was mostly seen in cases of acute and severe COVID-19 [17]. Menter et al. described placental pathology findings in five SARS-CoV-2 positive women delivering at term. They found features of malperfusion in all of the placentas examined, while villitis and intervillositis only in one case of acute maternal infection at the time of delivery [17]. Therefore, pregnant women with SARS-CoV-2 infection are not a homogenous group in terms of placental pathology. Placental changes associated with this infection differ depending on severity and timing of infection. Moreover, some placental changes may be associated with increased fetal risks as compared to others. The case presented suggests that SARS-CoV-2 associated inflammatory placental changes pose an especially elevated risk for the fetus. Maternal and fetal blood samples were not examined for the presence of anti SARS-CoV-2 antibodies or SARS-CoV-2 viral RNA. Vertical transmission can, therefore, not be entirely excluded as the cause of fetal death [11–13]. This should certainly be viewed as a limitation of this report despite postmortem fetal evaluation revealing no sign of fetal viral infection.

Histopathologic changes associated with SARS-CoV-2 infection of the placenta described in our case report would seriously hinder intervillous blood flow, disrupt the placental barrier, and cause inadequate exchange of nutrients, gases, and metabolic end products. These changes could explain the increased risks of fetal death described with maternal SARS-CoV-2 infection in several cohort studies [1, 7–10]. A single case does not allow drawing definitive conclusions on causality. It does, however, emphasize the need for larger prospective placental studies in SARS-CoV-2 infection during pregnancy which will eventually help to identify features of this infection that pose particularly increased risks to the developing fetus.

## Data Availability

Data are available on request through authors (e-mail of the corresponding author: miha.lucovnik@kclj.si).
